# Effect of synthesis time on plasmonic properties of Ag dendritic nanoforests

**DOI:** 10.1107/S2052252522002901

**Published:** 2022-04-02

**Authors:** Hung Ji Huang, Han-Wei Chang, Chia-Yen Lee, Ming-Hua Shiao, Yen-Ling Chiu, Pee-Yew Lee, Yung-Sheng Lin

**Affiliations:** a Department of Electra-Optical Engineering, National Formosa University, Yunlin 632301, Taiwan; b Department of Chemical Engineering, National United University, Miaoli 360302, Taiwan; c Department of Electrical Engineering, National United University, Miaoli 360302, Taiwan; d Taiwan Instrument Research Institute, National Applied Research Laboratories, Hsinchu 300092, Taiwan; e Department of Optoelectronics and Materials Technology, National Taiwan Ocean University, Keelung 202301, Taiwan; f PhD Program in Materials and Chemical Engineering, National United University, Miaoli 360302, Taiwan; g Institute of Food Safety and Health Risk Assessment, National Yang Ming Chiao Tung University, Taipei 112304, Taiwan

**Keywords:** Ag dendritic nanoforests, fluoride-assisted galvanic replacement reaction, surface-enhanced Raman scattering, surface plasmons, crystal morphology

## Abstract

The effects of synthesis time on the plasmonic properties of Ag dendritic nanoforests on Si substrate (Ag-DNF/Si) samples synthesized through the fluoride-assisted galvanic replacement reaction were investigated. The Ag-DNF/Si samples were characterized using scanning electron microscopy, energy-dispersive X-ray spectroscopy, reflection spectroscopy, X-ray diffraction and surface-enhanced Raman spectroscopy (SERS). The prolonged reaction time led to the growth of an Ag-DNF layer and etched Si hole array. SEM images and variations in the fractal dimension index indicated that complex-structure, feather-like leaves became coral-like branches between 30 and 60 min of synthesis. The morphological variation during the growth of the Ag DNFs resulted in different optical responses to light illumination, especially those of light harvest and energy transformation. The sample achieved the most desirable light-to-heat conversion efficiency and SERS response with a 30 min growth time. A longer synthesis time or thicker Ag-DNF layer on the Si substrate did not have superior plasmonic properties.

## Abstract 2

The morphological variation of longer-time synthesized or thicker Ag dendritic nanoforests on Si substrates did not have superior plasmonic properties. The prepared sample achieved the most desirable light-to-heat conversion efficiency and SERS response with an intermediated growth time.

## 1. Introduction

Plasmonic materials are used in numerous applications, including steam generation (Zhu *et al.*, 2018 ▸), plasmonic polymerase chain reaction (PCR) (Huang, Chiang *et al.*, 2020 ▸; Jeong *et al.*, 2018 ▸; Kim *et al.*, 2017 ▸; Roche *et al.*, 2017 ▸), plasmonic photocatalytic reaction (Huang, Wu *et al.*, 2020 ▸; Yang *et al.*, 2021 ▸; Agrawal *et al.*, 2021 ▸), surface-enhanced Raman spectroscopy (SERS; Ye *et al.*, 2008 ▸; Lai *et al.*, 2017 ▸; Shiao *et al.*, 2021 ▸; Litti *et al.*, 2021 ▸; Lin *et al.*, 2012 ▸; Jin *et al.*, 2021 ▸; Arabi *et al.*, 2021 ▸; Li, Wuethrich *et al.*, 2021 ▸; Peng *et al.*, 2021 ▸), solar cells (Jangjoy *et al.*, 2021 ▸; Ibrahim *et al.*, 2021 ▸; Heidarzadeh & Bahador, 2021 ▸) and light-assisted fuel cells (Lin *et al.*, 2015 ▸; Li, Miao *et al.*, 2021 ▸; Xu *et al.*, 2020 ▸). Two-dimensional plasmonic substrates are fabricated using top-down semiconductor processing or patterned fabrication (Lin *et al.*, 2012 ▸; Lai *et al.*, 2017 ▸), laser writing (Abou Khalil *et al.*, 2017 ▸) or the adhesion of bottom-up synthesized metal nanoparticles (Wang *et al.*, 2021 ▸; Liu *et al.*, 2020 ▸). The advantage of planar plasmonic substrates is that no recycling processes continue after use, but the disadvantage is that their single-layered structure limits the effects. Three-dimensional plasmonic substrates with a thick, artificially structured, metallic fixed layer have higher light energy conversion efficiency. Three-dimensional plasmonic substrates can be fabricated by stacking through top-down fabrication, laser writing (Huerta-Murillo *et al.*, 2017 ▸), atomic layer deposition in the empty spaces of removable templates (Chen *et al.*, 2014 ▸; King *et al.*, 2005 ▸; Bae *et al.*, 2011 ▸), bottom-up chemical synthesis of fractal structures (Ye *et al.*, 2008 ▸; Huang, Chiang *et al.*, 2020 ▸; Shiao *et al.*, 2021 ▸; Lin *et al.*, 2015 ▸; Huang, Chang *et al.*, 2020 ▸; Shiao, Lin, Huang *et al.*, 2018 ▸; Fang *et al.*, 2007 ▸; Kharisov *et al.*, 2015 ▸; Xu *et al.*, 2016 ▸; Lee *et al.*, 2017 ▸; Sun *et al.*, 2013 ▸; Choudhury *et al.*, 2021 ▸) and inclined deposition (Gao *et al.*, 2018 ▸; Sun *et al.*, 2020 ▸; Eiamchai *et al.*, 2020 ▸). The first three methods have the advantage of controllable fabrication but the disadvantages of high production cost and long processing time.

By contrast, chemically synthesized fractal structures are fabricated with low production costs and short processing times, enabling mass production and wide applications (Ye *et al.*, 2008 ▸; Huang, Chiang *et al.*, 2020 ▸; Huang, Chang *et al.*, 2020 ▸; Shiao, Lin, Huang *et al.*, 2018 ▸; Shiao, Lin, Zeng *et al.*, 2018 ▸; Shiao *et al.*, 2019 ▸). Chemically synthesized plasmonic materials typically present a complex fractal structure that is advantageous with a large surface area for chemical reaction; the complex network structure filters reactants or target materials. The tips and branches of lengthened metal leaves and twigs generate hot spots in the electromagnetic field or localized collective oscillation of free electrons under external light illumination (Huang, Wu *et al.*, 2020 ▸). Their low production cost, short production time, high production yield and high plasmonic response may make chemically synthesized fractal structures useful light energy converters.

Metal dendritic nanoforests (DNFs) are chemically synthesized fractal structures that can grow to tens of micrometres in thickness (Mandelbrot, 1982 ▸). The excellent plasmonic response of metal DNFs enhances the efficiency of light-assisted fuel cells (Lin *et al.*, 2016 ▸), bacterial inhibition (Huang, Chang *et al.*, 2020 ▸), SERS (Ye *et al.*, 2008 ▸; Shiao *et al.*, 2021 ▸) and light-to-heat energy conversion (Huang, Chiang *et al.*, 2020 ▸). Among the metals used, Ag has been demonstrated to have a notable enhancement factor (EF) for SERS (Ye *et al.*, 2008 ▸; Reed *et al.*, 2012 ▸; Han *et al.*, 2011 ▸; Zhao *et al.*, 2014 ▸; Liu *et al.*, 2014 ▸). Through adjustment of the initial Ag^+^ concentration, volume ratio of buffered oxide etching solution and duration (0.5–10 min) of the fluoride-assisted galvanic replacement reaction (FAGRR) for the growth of Ag-DNFs, an EF of 9.18 × 10^8^ was achieved for SERS for 4-mercapto­benzoic acid molecules (Shiao *et al.*, 2021 ▸).

If a thicker DNF layer is desired, a longer growth time may increase the layer thickness and thus its plasmonic applications. However, some Ag atoms with an excess of chemical potential on the metal DNF surface are unstable. Therefore, they can move away to be suspended and refixed to the metal DNF surfaces in a different place. This can lead to an unexpected surface morphology for the synthesized layer. In this study, various methods studied and characterized the effects of prolonged 120 min FAGRR synthesis of Ag-DNF/Si. In addition, the variations in the surface morphology of the Ag-DNF/Si and plasmonic enhancements of the reflection spectrum, light-to-heat energy conversion and SERS response were investigated.

## 2. Materials and methods

### 2.1. Preparation of Ag-DNF/Si substrates

Ag-DNF/Si samples were synthesized through FAGRR (Ye *et al.*, 2008 ▸; Huang, Chiang *et al.*, 2020 ▸; Huang, Chang *et al.*, 2020 ▸; Shiao, Lin, Huang *et al.*, 2018 ▸; Shiao, Lin, Zeng *et al.*, 2018 ▸; Shiao *et al.*, 2019 ▸). The F^−^ stabilizes the oxidation state Si^4+^ in solution, producing SiF_6_^2−^ and releasing four electrons. The generated electrons move to the rough surfaces of the Si or Ag, where Ag^+^ is reduced to Ag. The reactions can be expressed as follows:

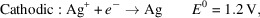




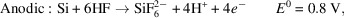




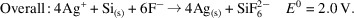




The synthesis of Ag-DNF/Si started with the ultrasonic washing of a 3.5 × 3.5 cm *n*-type Si substrate with acetone, methanol and deionized water for 5 min. The Si substrate was then dried using N_2_ spray for 5 min and heated in an oven at 120°C for 5 min. Next, the dried Si substrate was treated using a 10% HF solution for 20 s to remove the native oxide layer on the Si substrate. HF etching can also increase the roughness of the Si substrate and thus the adhesion of the synthesized Ag trees. Finally, the Si substrate was treated with a mixture comprising 24 ml reactant solution (8 ml 10% HF, 16 ml deionized water and 240 µl 1 *M* AgNO_3_) in a Teflon container measuring 5.5 cm in inner diameter and 4.8 cm in depth. The synthesis times were 5, 15, 30, 60 or 120 min. The synthesized Ag-DNF/Si were washed three times using deionized water, and the samples were dried with N_2_ spray and then incubated at 120°C for 5 min to obtain the Ag-DNF/Si substrates.

### 2.2. Characterization

The material properties of the synthesized Ag-DNF/Si substrates were characterized through cold-field emission scanning electron microscopy (SEM; SU-8010, Hitachi, Tokyo, Japan) and X-ray diffraction (XRD; D8 Discover, Bruker, Billerica, USA). An ultraviolet–visible reflection spectrophotometer (UV-3101PC, Shimadzu, Kyoto, Japan) with a spherical light integrator was used to measure the reflection spectra of the samples. High-performance Brunauer–Emmett–Teller (BET) surface area analysis and a pore size analyzer [Micro 100C, 3P Instruments GmbH & Co. KG, Germany (Scherdel *et al.*, 2010 ▸)] were used to measure the multilayer adsorption on the surface of the samples.

### 2.3. Image analysis

The fractal dimension is an index that characterizes a fractal pattern by quantifying its complexity as the ratio of detail change to scale change (Mandelbrot, 1982 ▸), as illustrated in the equation below. The dimension (*D*) can be calculated as the change in the number of self-similar structures (*N*) with respect to the magnification factor (*r*),

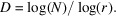




### 2.4. Light-to-heat energy conversion experiments

Light-to-heat energy conversion experiments were conducted using a 90 W halogen lamp illuminating the Ag-DNF/Si samples inside a circular glass petri dish under ambient conditions. The temperature of the Ag-DNF/Si under illumination was measured using a thermal imager and recorded.

### 2.5. SERS analysis

Rhodamine 6G (R6G) SERS was conducted using a Raman spectroscope with a 632.8 nm light source (UniDRON, UniNanoTech, South Korea). For the measurements, the samples were dipped in 10 ml of 10^−6^ *M* R6G solution for 30 min in a glass petri dish and dried through incubation at 37°C for 24 h.

## 3. Results

**Figure 1:**
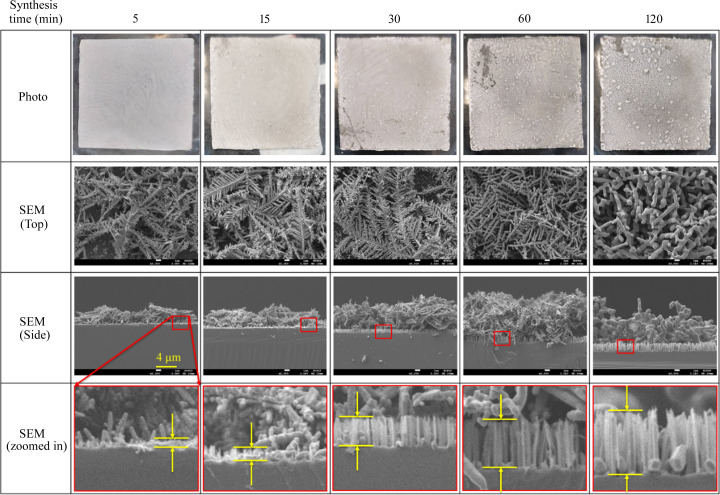
Top-view photographs and SEM images (top and side views) of Ag–dendritic nanoforest (DNF)/Si samples fabricated with various synthesis times.

The photographs and SEM images in Fig. 1 ▸ present the Ag-DNF/Si substrates synthesized for various durations. The area of the 5 min sample was coated with 3.37 µm-thick white Ag-DNFs, measured by SEM. The measured thickness was 3.82 and 5.67 µm for the 15 and 30 min samples, respectively. SEM revealed feather-like leaves compacted in a dense brushwood forest structure for 5–30 min syntheses. The feather-like leaves became thick coral-like branches after longer synthesis durations. After 60 min, the thickness of the Ag-DNF layer was 9.59 µm. However, the thickness decreased to 8.15 µm for the sample synthesized for 120 min.

Side-view SEM revealed that the Si substrate was etched to create an Si nanopillar array and tiny Ag nanocrystals, resembling the results obtained by Qiu *et al.* (2005 ▸). The nanopillar array etched on the Si substrate had a more uniform thickness than the deposited Ag-DNFs.

**Figure 2:**
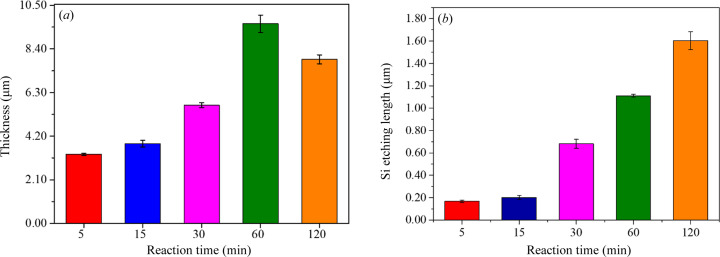
(*a*) Thickness of Ag-DNF layer and (*b*) Si etching depth for various synthesis times.

The average thicknesses of the synthesized DNF layer and the etched Si nanopillar array depth are depicted in Figs. 2 ▸(*a*) and 2 ▸(*b*), respectively. The SEM images indicate that the average thickness of the DNFs was the highest for the 60 min synthesis, whereas the depth of the Si hole array continued to increase over time. The samples exhibited differences in morphology, the thickness of the DNF layer and the depth of the Si nanopillar array. Experimental characterization was used to investigate the effects of these differences.

The fractal dimension *D* (Mandelbrot, 1982 ▸) was determined for the Ag-DNF/Si samples. Table 1 ▸ presents the fractal dimension indices of the samples from the top-view 5000× magnification SEM images in Fig. 1 ▸; *D* decreased as synthesis durations increased. *D* was significant for 5 (1.729) to 30 min (1.725) syntheses but decreased by 60 min (1.579). The variation in *D* values indicates that the feather-like leaves observed for 30 min syntheses were becoming thick coral-like branches by 60 min.

**Figure 3:**
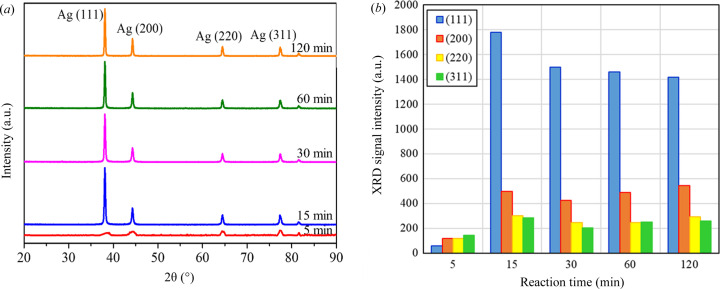
(*a*) XRD data of various Ag-DNF/Si samples. (*b*) XRD peak intensities of crystal faces of synthesized Ag-DNFs.

Fig. 3 ▸(*a*) presents the XRD results for the samples. The Ag-DNFs had a face-centered cubic (f.c.c.) crystal structure with cell dimensions of 4.08 Å (Emsley, 1998 ▸). The f.c.c. structure had Ag(111), (200), (220) and (311) crystal faces (Godipurge *et al.*, 2016 ▸). The XRD signal of Ag(111) was dominant. The detected XRD peak intensity of the various crystal faces, as shown in Fig. 3 ▸(*b*), presented a sharp signal peak for Ag (111). The change in peak heights was minimal between 15 and 30 min of growth. The XRD data did not contain signals indicating the generation of AgO, Si or SiO_2_. Therefore, the synthesized DNFs were Ag thoroughly covering the Si substrate.

**Figure 4:**
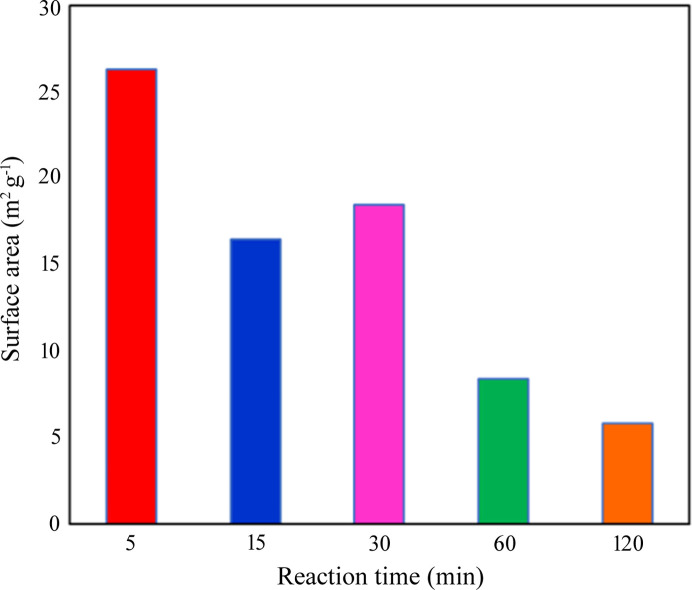
Specific surface areas of Ag-DNF samples with various synthesis times.

BET analysis is sensitive to multilayer surface adsorption (Scherdel *et al.*, 2010 ▸). The specific surface areas of the 15, 30, 60 and 120 min samples were 16.5, 18.5, 8.4 and 5.8 m^2^ g^−1^, respectively (Fig. 4 ▸). The 5 min sample had the largest surface area: 26.3 m^2^ g^−1^. However, the 5 min Ag-DNFs were shorter than the 30 min Ag-DNFs. Therefore, the 30 min Ag-DNFs had more molecules fixed on the surface than the 5 and 15 min Ag-DNFs. The specific surface area decreased as the synthesis time increased, which resembles the result obtained by Qiu *et al.* (2005 ▸).

**Figure 5:**
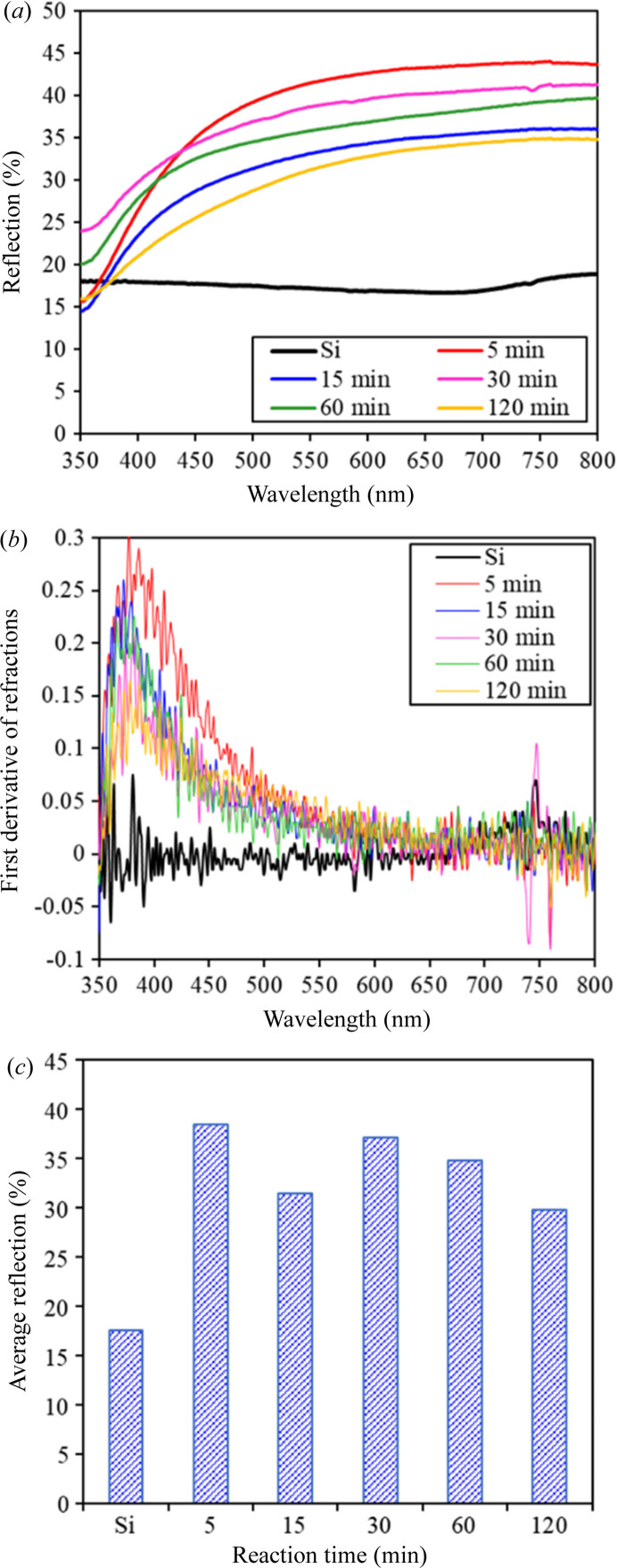
Si substrate and Ag-DNF/Si samples with various synthesis times: (*a*) reflection spectra, (*b*) first-derivative spectrum and (*c*) average reflection in spectra (350–800 nm).

Fig. 5 ▸(*a*) shows that the synthesized Ag-DNF/Si samples prominently reflected light with wavelengths longer than 450 nm. Light from direct reflection and the multiple scattering processes is measured inside an optical integrator. As shown in Fig. 5 ▸(*b*), the first derivative of the reflections of various samples peaked at 380–390 nm, leading to the typical optical plasma frequency for Ag at a wavelength of 400 nm (Ordal *et al.*, 1983 ▸; Han *et al.*, 2008 ▸). The dense leaves and branches of Ag-DNFs reduce the optical reflection of incident light. Light travels through gaps between the sharp leaves of Ag branches and is trapped after multiple scattering in the deep forest. Therefore, the Ag-DNFs grown for various synthesis times should present differences in light reflection and absorption. Also, a deeply etched Si nanopillar array (Arzumanyan *et al.*, 2017 ▸; Fan *et al.*, 2021 ▸; Cheng *et al.*, 2021 ▸; Uddin *et al.*, 2021 ▸; Omar *et al.*, 2021 ▸), as shown in the side-view SEM images in Fig. 1 ▸, should result in high light absorption. Therefore, the combination of an Ag-DNF layer and an Si nanopillar array layer results in an oscillating reaction-time dependence in the reflected light spectra. The average reflections of the Si substrate and Ag-DNF/Si samples in the acquired spectrum are presented in Fig. 5 ▸(*c*). The changes in reflection affect the potential plasmonic applications and are discussed in the following sections.

**Figure 6:**
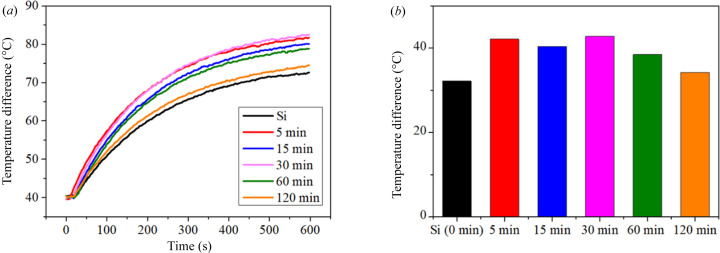
Plasmonic light-to-heat energy conversion in water-heating experiments with various samples. (*a*) Temperature increases from 40°C. (*b*) Temperature increases after water-heating experiments.

As shown in Fig. 6 ▸, light-to-heat energy conversion experiments were used to determine the plasmonic response of the Ag-DNF/Si substrates in the near-field zone. An infrared (IR) imager measuring the sample temperature can collect IR light from a halogen lamp; therefore, the starting temperature for measurement in this study was approximately 40°C. The halogen lamp required tens of seconds to turn on entirely and 20 min to stabilize; thus, a low-temperature period occurred at the beginning of all experiments. The temperature of the Ag-DNF/Si increased exponentially under light illumination, as shown in Fig. 6 ▸(*a*). The 30 min sample had the most significant temperature increase (42.8°C), as shown in Fig. 6 ▸(*b*). Si can absorb light and convert it into heat, and the temperature of the plain Si substrate also increased by approximately 32.2°C. The thickness of the Ag-DNF layer from the 5 min synthesis was 3.37 µm. Most lights were absorbed by the dense Ag-DNF layer and plasmonically converted into heat. Therefore, the Ag-DNF layer exhibited an efficient plasmonic response in light-to-energy conversion in the near-field zone.

**Figure 7:**
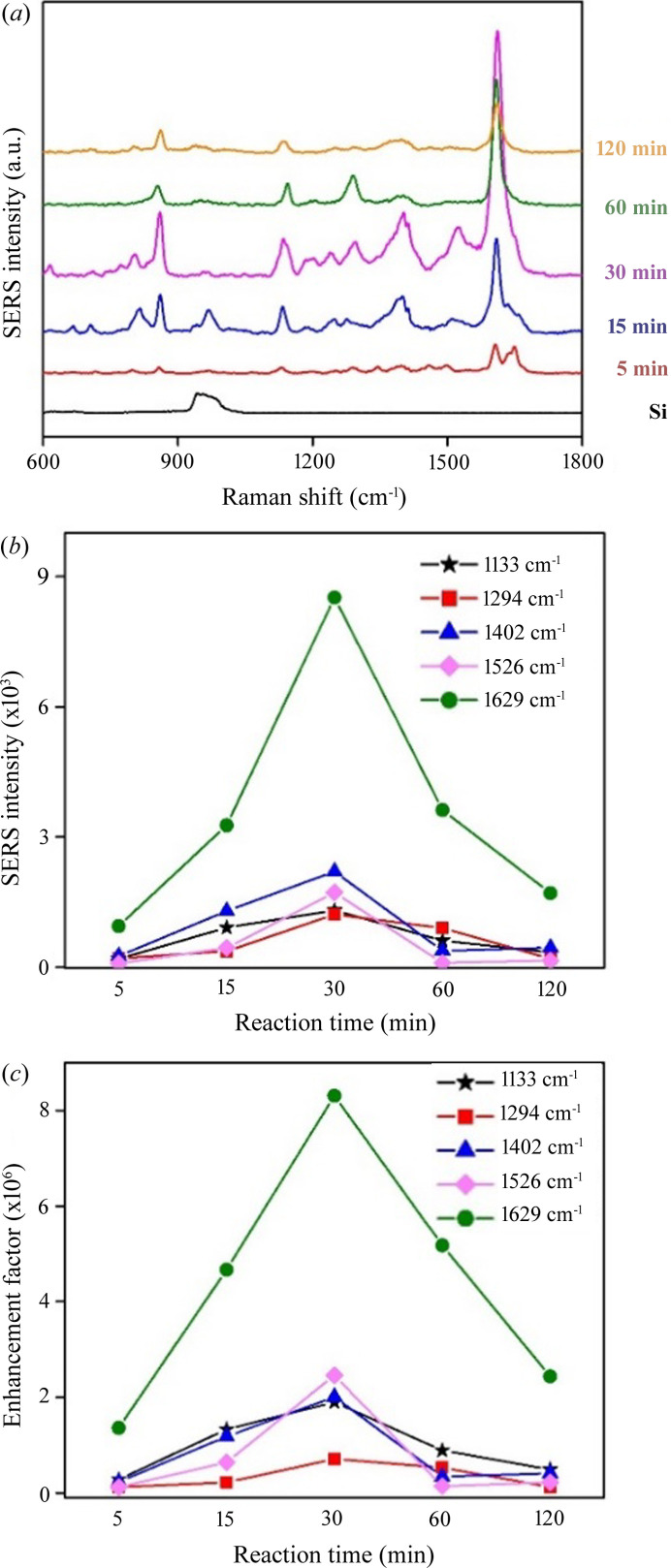
(*a*) Raman spectra of aqueous rhodamine 6G (10^−6^ *M*) solution for Ag-DNF/Si samples. (*b*) Variation of SERS intensity peak heights and (*c*) enhancement factors among samples.

As shown in Fig. 7 ▸(*a*), the prominent peaks of the SERS responses to R6G of the Ag-DNF/Si samples were 772 cm^−1^, indicating out-of-plane bending; 1185 cm^−1^, indicating in-plane C—H bending; 1311 cm^−1^, indicating C—O—C stretching; and 1363, 1510, 1575 and 1650 cm^−1^ indicating aromatic C—C stretching (Kosović *et al.*, 2015 ▸). The intensities of the Raman peaks increased with the synthesis time up to 30 min but decreased with more prolonged synthesis. Thus, the 30 min sample exhibited the most substantial plasmonic response under light illumination, a result similar to that of the light-to-heat energy conversion experiments (Fig. 6 ▸).

## 4. Discussion

The prolonged reaction led to various growth of Ag-DNF layers and a deeply etched Si nanopillar array (Fig. 1 ▸). The surface morphology of the synthesized Ag DNFs changed as the reaction continued beyond 30 min. These changes resulted in variations in the plasmonic response to external light illumination.

Ag has a higher electric potential than Si. Therefore, the *e* ^−^ generated from the reaction and etching of Si atoms by HF flowed to the Ag^+^ or deposited Ag on the surface of the substrate. The *e* ^−^ accumulated on the Ag nanoparticles, expelling F^−^ in water. This resulted in the etching of Si only in spaces without Ag nanoparticles and a deep Si nanopillar array. The density of Ag^+^ ions in the upper layer was high, and the Ag^+^ was reduced to Ag and stacked as leaves and branches. As a result, the average thickness of the Ag-DNFs was the highest after 60 min of synthesis [Fig. 2 ▸(*a*)], despite the etching of Si continuously providing *e* ^−^ and the etching depth of the Si hole array increasing throughout the synthesis [Fig. 2 ▸(*b*)].

The Ag DNFs appearance changed with the reaction time (in Fig. 1 ▸). The random walk of Ag^+^ resulted in the feather-like Ag leaves being reduced to Ag over time. The XRD peak intensity was low despite the thickness of the Ag-DNFs layer being 3.32 µm after 5 min of reaction (Fig. 3 ▸). Fixed Ag atoms dissolved in the solution or oxidized to become Ag^+^ again and then were converted into the coral-like branches observed for synthesis times longer than 30 min. Dissolved Ag has a higher density than H_2_O and can diffuse to places near the bottom of the Ag branches. Therefore, Ag atoms traveled to places with lower chemical energy and higher stability. The tiny feather-like Ag nano-leaves gradually disappeared with synthesis durations greater than 30 min. The *D* value of the sample synthesized for the shortest time (5 min) was the largest (1.729; Table 1 ▸), and the *D* value of the 60 min sample was much lower than that of the 30 min sample. The variation in the fractal dimension index indicated the transformation of feather-like leaves into coral-like branches.

The XRD data (Fig. 3 ▸) indicated that the peak intensities of all the crystal faces had vastly changed by 15 min of growth and settled gradually with slight variations after because of the most random growth of fractal structures with large surface areas. A combination of XRD (see Fig. 3 ▸) and BET measurements (see Fig. 4 ▸) was used to characterize the transformation of the Ag-DNFs. The 5 min Ag-DNF sample had the largest specific surface area and XRD peaks owing to the number of detectable crystal surfaces.

The variations in the surface morphology during growth of the Ag-DNFs resulted in different optical responses to light illumination, especially light harvest and energy transformation. The reflection spectrum indicated a broadband homogeneous partial reflection of light with wavelengths longer than 500 nm [Fig. 5 ▸(*a*)]; 60%–70% of visible light [see Fig. 5 ▸(*c*)] was absorbed. The trapped light was partially transformed into heat or induced SERS; thus, the material may be suitable for rapid plasmonic PCR (Huang, Chiang *et al.*, 2020 ▸; Jeong *et al.*, 2018 ▸; Kim *et al.*, 2017 ▸; Roche *et al.*, 2017 ▸) or chemical/biological detection (Shiao *et al.*, 2021 ▸; Litti *et al.*, 2021 ▸; Lin *et al.*, 2012 ▸; Lai *et al.*, 2017 ▸; Jin *et al.*, 2021 ▸; Arabi *et al.*, 2021 ▸; Li, Wuethrich *et al.*, 2021 ▸; Peng *et al.*, 2021 ▸). We conducted a light-to-heat experiment (Fig. 6 ▸) and R6G SERS (Fig. 7 ▸) to evaluate the plasmonic responses of the samples.

Plasmonic energy transformation (*i.e.* light reflection, light-to-heat energy transformation and SERS) can be roughly interpreted as a three-step process. Step 1 is the light-induced generation of a strong electromagnetic field or (localized) surface plasmons. Step 2 is the conversion of the strong electromagnetic field or plasmonic energy into the target material, *e.g.* R6G molecules. Step 3 is the inverse transformation or release of the strong electromagnetic field or plasmons to the far-field regime. In the light-to-heat energy transfer process, plasmon-induced quantum hot charge carriers decay in plasmon–phonon interactions or joule heating by creating currents of collective oscillating electrons that, combined, increase the temperature of the microenvironment (Jeong *et al.*, 2018 ▸; Kim *et al.*, 2017 ▸; Roche *et al.*, 2017 ▸; Huang, Wu *et al.*, 2020 ▸; Huang, Chang *et al.*, 2020 ▸) in step 2 of plasmonic energy transformation.

In SERS, the plasmon–material interaction on metal nanomaterials in step 2 occurs through both short-range chemical enhancement and long-range electromagnetic enhancement (Wang *et al.*, 2013 ▸; Pham *et al.*, 2017 ▸). The long-range electromagnetic enhancement is caused by hot spots (*i.e.* strong electromagnetic fields) of localized surface plasmon resonance under the excitation of light on or near the surface of metallic nanoparticles (Pham *et al.*, 2017 ▸; Jones *et al.*, 2011 ▸). The chemical enhancement in step 2 relies on the conduction of the light-generated high-energy (*i.e.* hot) electrons on the metal surface to the target molecules. For short-range chemical enhancement in step 2, the target molecules must contact the surface of the SERS sensors. The BET measurements (Fig. 4 ▸) suggest that the Ag-DNF layer from the 5 min synthesis had the largest specific surface area. However, the Ag-DNF layer from the 30 min synthesis was thicker and able to adsorb more target molecules (R6G in this work). The 60 min Ag-DNFs had a weaker SERS response due to the much smaller specific surface area and plasmon resonance response.

The nano-features of Ag and Si branches enhance light absorption and plasmonic energy conversion (Jeong *et al.*, 2018 ▸; Kim *et al.*, 2017 ▸; Roche *et al.*, 2017 ▸; Huang, Wu *et al.*, 2020 ▸; Huang, Chang *et al.*, 2020 ▸; Cheng *et al.*, 2021 ▸). The transition of the Ag-DNFs from feather-like leaves to coral-like branches led to decreased effectiveness of the nanoantennas and metal nanogaps. The plasmonic hot spots used for high-efficiency plasmon generation and plasmon-to-light conversion tend to be induced on nanoantennas and in metal nanogaps. However, the coral branches of the Ag-DNFs from the 60 and 120 min syntheses had few nanostructures suited to the generation of plasmon hot spots. The light-induced generation of hot spots occurs in the near-field. The plasmonic hot spots induced in step 1 are light energy localized in tiny areas, enduring the high-efficiency step 2 plasmon–material interaction. The reverse of the optical process in step 1 is step 3. Therefore, the SERS-generated light in step 2 in a hot spot can be efficiently extracted and acquired in the far-field.

The combined effects of the specific surface area, light absorption inside the Ag branches in the DNFs and plasmonic hot spots in the complex branched structures resulted in the reflection spectrum, light-to-heat energy conversion and R6G SERS response synthesized Ag-DNFs having a dependence on the reaction time. The etched Si nanopillars were also responsible for trapping incident light and resulted in light-to-heat conversion. The conversion efficiency and SERS response of the sample with a 30 min growth time were the most desirable. A longer synthesis duration or thicker Ag-DNF layer on the Si substrate did not have superior plasmonic characteristics.

Chemically synthesized Ag-DNFs have a desirable three-dimensional structure with the benefits of lower fabrication costs and shorter fabrication time than structures fabricated through semiconductor methods. In addition, such Ag-DNFs have potential in light-harvesting, bacterial inhibition (Huang, Chang *et al.*, 2020 ▸), SERS [9,24] and rapid plasmonic PCR (Huang, Chiang *et al.*, 2020 ▸). However, careful manipulation of the synthesis process is required to optimize performance, for example, controlling the synthesis time as demonstrated in this study.

## 5. Conclusions

A prolonged reaction time allowed the growth of an Ag-DNF layer and an etched Si hole array. The drop in the fractal dimension index (*D*) after 30 min can be attributed to the transformation of feather-like leaves into coral-like branches. The variation of the surface morphology during the growth of the Ag-DNF resulted in differences in the optical responses to light illumination, especially the light harvest and energy transformation. The nanofeatures of the Ag and Si branches effectively enhanced light absorption and plasmonic energy conversion. With their high specific surface area, the materials absorbed light in the Ag branches, and plasmonic hot spots formed due to the complex branched structures. The reflection spectra, light-to-heat energy transformation and SERS response of the Ag-DNF/Si were dependent on the reaction time. The etched Si nanopillars also trapped incident light and resulted in light-to-heat conversion. The optimized light-to-heat conversion efficiency and SERS response were obtained for the sample with a 30 min growth time. A longer synthesis time or thicker Ag-DNF layer on the Si substrate did not achieve superior plasmonic properties.

## Figures and Tables

**Table 1:** Fractal dimension index (*D*) of Ag-DNF/Si substrates synthesized for various times

Reaction time (min)	5	15	30	60	120
*D*	1.729	1.726	1.725	1.671	1.579
Δ*D*	–	0.003	0.001	0.054	0.092
